# Comparison of Total Corneal Astigmatism between IOLMaster and Pentacam

**DOI:** 10.1155/2022/9236006

**Published:** 2022-07-08

**Authors:** Xiaochun Li, Xiaoguang Cao, Yongzhen Bao

**Affiliations:** ^1^Department of Ophthalmology, Peking University People's Hospital, Eye Diseases and Optometry Institute, Beijing Key Laboratory of Diagnosis and Therapy of Retinal and Choroid Diseases, College of Optometry, Peking University Health Science Center, 11 Xizhimen South Street, Xicheng District, Beijing 100044, China; ^2^Department of Ophthalmology, Peking University International Hospital, 1 Shengmingyuan Road, Life Science Park, Changping District, Beijing 102206, China

## Abstract

**Purpose:**

To compare the total corneal astigmatism (TCA) measured by IOLMaster 700 and Pentacam and to investigate the consistency of corneal keratometry (CK) measured by IOLMaster and Pentacam.

**Methods:**

Cataract patients were retrospectively enrolled in March and April, 2021. Retrospective analysis was performed on those patients with binocular and monocular CK measured by IOLMaster and Pentacam.

**Results:**

A total of 102 patients (204 eyes) were included, 64 of whom were female (62.75%). The flat (K1) and steep (K2) CK of anterior corneal surface (ACS) and flat (TK1) and steep (TK2) of total cornea measured with IOLMaster 700 were 44.16 ± 1.60 D, 45.09 ± 1.68 D, 44.12 ± 1.62 D, and 45.14 ± 1.69 D, respectively. Those measured with Pentacam were 44.31 ± 1.57 D, 45.22 ± 1.65 D, 44.15 ± 1.67 D, and 45.19 ± 1.82 D, respectively. The astigmatism of ACS and TCA were −0.94 ± 0.63 D and −1.02 ± 0.69 D (*p* < 0.01) in the IOLMaster group and −0.90 ± 0.59 D and −1.05 ± 0.81 D in the Pentacam group, respectively (*p* < 0.01). TCA measurement results of IOLMaster and Pentacam were consistent (Pearson = 0.710, *p* < 0.01), and there was no significant difference (*p* = 0.591).

**Conclusions:**

Total corneal astigmatism measured by IOLMaster was consistent with that measured by Pentacam. The difference between the astigmatism of anterior corneal surface and total cornea was detected in the measurement of IOLMaster and Pentacam, respectively.

## 1. Introduction

With the application of toric and presbyopia-correcting intraocular lens (IOL, toric IOL and PC-IOL), precise assessment of preoperative corneal astigmatism (CA) has played an increasingly important role in the past twenty years [1–4]. The larger the CA, the poorer the postoperative visual acuity at all distances after implantation of either monofocal or multifocal IOLs [5, 6]. Improvements of postoperative astigmatism, such as toric IOL, clear corneal incision location, and peripheral corneal relaxing incisions, were developed. All of these were based on accurate preoperative CA measurement.

The keratometer, as a standard device for corneal keratometry (CK) measurement, has been widely used in clinic practice for many years. The problem is that the CA measurement results are different with different types of orthokeratology devices. The main limitation of CA measurements currently available is that they are limited to the anterior corneal surface (ACS) [7–11]. Previous studies have shown that CA of posterior corneal surface (A-PCS) cannot be ignored in at least some eyes [12]. In addition, the astigmatism of ACS (A-ACS) scatter axis is quite different from that of the A-PCS axis, resulting in different total CA (TCA).

The Pentacam Scheimpflug imaging system (OCULUS, Wetzlar, Germany) has been introduced into the clinic, mainly using the biometry measurement of corneal morphology [13, 14]. In many hospitals, the SimK of ACS provided by Pentacam is used to calculate the power of IOLs and the total CK (TCK), named Total Corneal Refractive Power (TCRP), also used for the choice of toric or PC-IOLs. Compared with the models and nomograms based on statistical measurement, Pentacam shows greater advantages in compensating for outliers [13].

IOLMaster 700 (Carl Zeiss Meditec, Germany) is the upgraded version of IOLMaster 500, which is widely used for the measurement of axial length (AL) and CK in cataract patients and others [15, 16]; TCK is also available recently [17–19]. It is now possible to measure the posterior corneal surface directly with SS-OCT and apply the formula on IOLMaster and Z-CALC, without changing the original clinic workflow.

TCA measured by IOLMaster and Pentacam might be different due to different principles and different measurement regions [20–22]. Comparison of TCK values obtained by IOLMaster, Pentacam, and other different devices on the same eye can provide clues to understand the trends of current corneal power measurement systems. Therefore, the primary purpose of this study was to investigate the consistency and correlation between IOLMaster and Pentacam of TCA measurements. The secondary objective was to compare A-ACS and TCA of TCK obtained by IOLMaster and Pentacam.

## 2. Methods

### 2.1. Sampling

The study was approved by the Human Research Ethics Committee of Peking University People's Hospital and adhered to the guidelines of the Declaration of Helsinki. Written informed consent was obtained from each subject after explaining the nature of this study. Consecutive patients diagnosed with cataract were retrospectively enrolled in March and April, 2021. The inclusion criteria were as follows: cataract patients with binocular biometry measurement obtained with IOLMaster 700 and Pentacam. Exclusion criteria for this study: corneal operation or corneal opacity (such as corneal leukoplakia, except cornea arcus senilis).

### 2.2. Data Acquisition

CK was measured using the same program of IOLMaster and Pentacam. In accordance with the user guidelines of each device, effective measurements (measurement quality check list was ok for Pentacam and three K readings difference would be less than a quarter diopter for IOLMaster) were used in the final analysis. The measured diameter of the central cornea is 2.5 mm for anterior and total CK in IOLMaster 700 and 3 mm for anterior and 4 mm for total CK in Pentacam. The software used was version 1.22r01 in Pentacam and version 1.88.1.64861 in IOLMaster 700. All measurements were performed in a semidark room. The subjects were asked to place their chin on the chin rest and press the forehead against the forehead strap. The eye was then aligned to the corneal topographic axis by using a central fixation light or target. The subjects were instructed to perform a complete blink before each measurement. All axis values more than 90° were recalculated by minus 180. For example, one axis value was 101° and recalculated as -79°.

### 2.3. Vector Analysis of Astigmatism

Vector analysis was used to compare CA values from these two devices. The astigmatism values were decomposed into two perpendicular components as follows: *X* = *A*∗cos(2*α*); *Y* = *A*∗sin(2*α*) [23–25]. And *J*0 = −(*A*/2)∗cos(2*α*); *J*45 = −(*A*/2)∗sin(2*α*). The corrected astigmatism values were calculated as follows: *α*va = arctan([Y1-Y2]/[X1-X2]), Ava = [Y1-Y2]/sin*α*va, where *X*, *X*1, and *X*2 are the cardinal component, *Y*, *Y*1, and *Y*2 are the oblique component, *A* and Ava are the astigmatism magnitude in diopters, and *α* and *α*va are the astigmatism axis in degrees; Ava is the corrected astigmatism with vector analysis.

### 2.4. Statistical Analysis

Statistical analyses were performed using commercial software (SPSS for Windows, Version 20.0; SPSS Inc., Illinois, USA). A *p* value of less than 0.05 was considered to be statistically significant. The Kolmogorov-Smirnov test was used to assess data normality. Based on the data normality test result, paired two-tailed *t*-test analysis and related sample nonparametric test were utilized to compare CA values among these two devices. The sample size for normality test is 204 and 102 for each eye. And the normality test was run as one sample in nonparametric test with automatic mode.

## 3. Results

204 eyes of 102 patients were included in the final study. Demographics of the study population are summarized in Table 1.

CK and CA obtained using IOLMaster and Pentacam are listed in Table 2.

The Kolmogorov-Smirnov test result demonstrated that not only the flat keratometry (K1) and the steep keratometry (K2) of anterior corneal surface but also the flat (TK1) and steep (TK2) of total cornea, measured with IOLMaster and Pentacam, passed the test of normality (all *p* > 0.05). However, the values of astigmatism did not pass the normality test. And the related sample nonparametric test was used to compare the difference of astigmatism.

**Figure 1:**
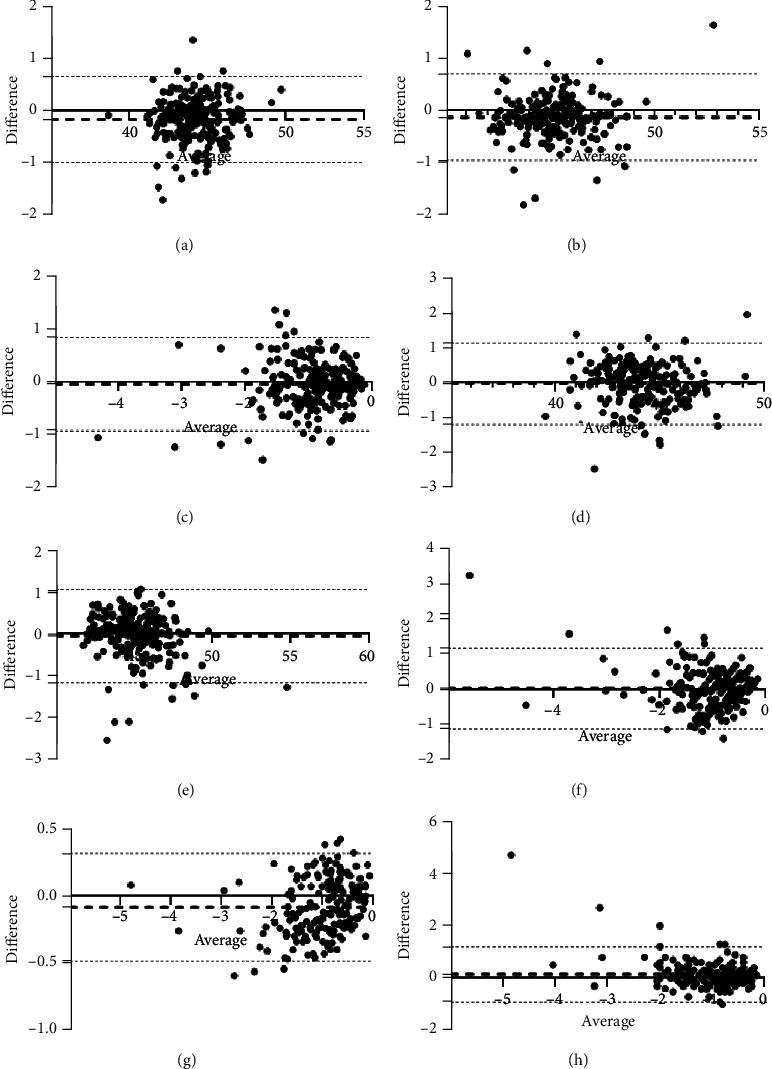
Bland–Altman plot shows consistency in the corneal keratometry and astigmatism (unit diopters (D) for the *x*-axis). (a) K flat of anterior corneal surface (K1) between IOLMaster and Pentacam. (b) K steep of anterior corneal surface (K2) between IOLMaster and Pentacam. (c) Astigmatism of anterior corneal surface (A-ACS) between IOLMaster and Pentacam. (d) K flat of total cornea (TK1) between IOLMaster and Pentacam. (e) K steep of total cornea (TK2) between IOLMaster and Pentacam. (f) Astigmatism of total cornea (TCA) between IOLMaster and Pentacam. (g) Astigmatism of anterior corneal surface (A-ACS) and total cornea (TCA) measured by IOLMaster. (h) Astigmatism of anterior corneal surface (A-ACS) and total cornea (TCA) measured by Pentacam.

A-ACS and TCA measured with IOLMaster were −0.94 ± 0.63 D@2.16 ± 59.94° and −1.02 ± 0.69 D@1.21 ± 64.34°; those measured with Pentacam were −0.90 ± 0.59 D@1.88 ± 50.13° and −1.05 ± 0.81 D@2.52 ± 55.61°. Those of A-ACS and TCA measured with IOLMaster and Pentacam for the right eyes were −0.94 ± 0.71 D@5.21 ± 58.33°, −1.02 ± 0.78 D@5.18 ± 63.20°, −0.88 ± 0.59 D@−1.95 ± 51.13°, and −1.07 ± 0.95 D@2.50 ± 54.45°. And those for the left eyes were −0.94 ± 0.53 D@−0.88 ± 61.64°, −1.03 ± 0.58 D@−2.76 ± 65.54°, −0.93 ± 0.58 D@5.71 ± 49.07°, and −1.02 ± 0.63 D@2.53 ± 57.00°. There were significant differences between A-ACS and TCA, measured with not only IOLMaster (*p* < 0.01) but also Pentacam (*p* < 0.01). And those were similar for the right eyes (*p* < 0.01 and *p* < 0.01) and the left eyes (*p* < 0.01 and *p* < 0.05). The differences between the astigmatism values measured with IOLMaster and Pentacam were not significant, either A-ACS (−0.04 ± 0.45 D, *p* = 0.307) or TCA (0.02 ± 0.58 D, *p* = 0.871). Those of A-ACS and TCA for the right eyes (−0.06 ± 0.45 D, *p* = 0.480; 0.05 ± 0.62 D, *p* = 0.652) and the left eyes (−0.01 ± 0.45 D, *p* = 0.467; −0.01 ± 0.54 D, *p* = 0.860) were similar.  Figure 1 shows the Bland–Altman plots to compare keratometric and astigmatic values with the mean difference and the upper and lower 95% LoA graphed between IOLMaster and Pentacam and visualized the consistency between the devices.

**Figure 2:**
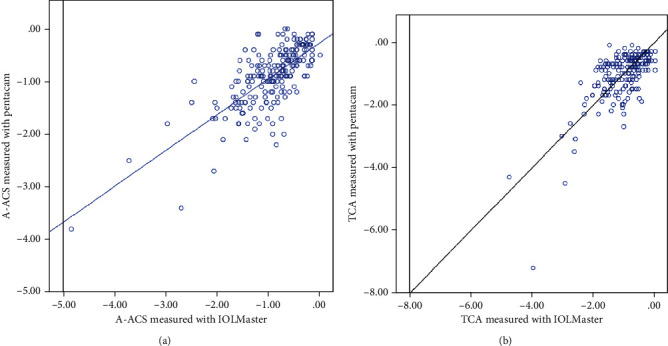
Correlation of corneal astigmatism between the corneal keratometry measured with IOLMaster and Pentacam (unit diopters (D)). (a) Correlation of anterior corneal surface astigmatism. A-ACS: corneal astigmatism of anterior corneal astigmatism. (b) Correlation of total corneal astigmatism. TCA: total corneal astigmatism.

Pearson analysis revealed the significant correlation between A-ACS and TCA, measured both in IOLMaster and Pentacam (Pearson = 0.956, *p* < 0.01; Pearson = 0.751, *p* < 0.01, respectively). Comparing the measurement results from the two devices, the correlations were significant in both A-ACS (Pearson = 0.729, *p* < 0.01) and TCA (Pearson = 0.710, *p* < 0.01), shown as Figure 2.

After vector analysis, the correct difference between A-ACS and TCA measured with IOLMaster was 0.20 ± 0.11 D, and that of Pentacam was 0.39 ± 0.47 D (*p* < 0.01). Those for the right eyes were 0.20 ± 0.11 D and 0.42 ± 0.59 D (*p* < 0.01) and for the left eyes were 0.20 ± 0.11 D and 0.37 ± 0.29 D (*p* < 0.01). Moreover, after vector analysis, the correct difference between the values got from IOLMaster and Pentacam was 0.42 ± 0.33 D of A-ACS and 0.56 ± 0.45 D of TCA (*p* < 0.01). Those for the right eyes were 0.40 ± 0.31 D and 0.55 ± 0.46 D (*p* < 0.01) and for the left eyes were 0.45 ± 0.35 D and 0.57 ± 0.44 D (*p* < 0.01). For vector components, Pearson analysis revealed the significant correlation for *J*0 and *J*45 of A-ACS between IOLMaster and Pentacam (Pearson = 0.849, *p* < 0.01; Pearson = 0.732, *p* < 0.01, respectively) and also of TCA between the devices (Pearson = 0.762, *p* < 0.01; Pearson = 0.638, *p* < 0.01, respectively).

## 4. Discussions

With the popularity of toric and PC-IOL in ophthalmology clinic, accurate CA measurement has an increasing influence on the postoperative refractive results. The accurate preoperative biometric measurement, especially CA, is the determination factor for the final toric IOL power and the meridian of IOL alignment required by all toric IOL calculators. Moreover, it is also a very important factor to help us to make the decisions, such as whether to use PC-IOL or not and what exact model to use. Besides the different measurement results due to the different keratometry and corneal topography devices, the curvature of posterior corneal surface, known as TCK, might also be responsible for refractive errors. One of the purposes of this study was to evaluate the TCK measurement accuracy by IOLMaster, a new method introduced recently. It is compared with Pentacam, an earlier method which has been used in clinic for many years to evaluate its consistency. Previous study had reported that Pentacam had large ACD values than IOLMaster [26]. Besides the difference of measuring mode, the measuring diameters also are different [27–29].

This study revealed good consistency of TCK measurement results by IOLMaster and Pentacam, similar with other study [27]. As Pentacam is an earlier device to measure TCK, good consistency between the two devices indicated that IOLMaster could be used as another method to obtain accurate cornea curvature and astigmatism to guide the usage of toric and PC-IOLs. As the two devices use different measurement principles and areas, minor differences were found in this study. The TCK value measured by IOLMaster is slightly less than that measured by Pentacam. After the vector analysis, the correction of total cornea astigmatism was quite similar.

IOLMaster has been regarded as a reliable method for measuring anterior corneal curvature in clinical practice. This study showed that there was no significant difference between A-ACS measured with IOLMaster and Pentacam, although the measurement results by IOLMaster were slightly higher than those by Pentacam. This is consistent with some previous studies [30–32]. This may be due to the relatively smaller corneal diameter measured by IOLMaster (2.5 mm) than that by Pentacam (3.0 mm or 4.0 mm) [33]. The mean difference of A-ACS between these two devices was only 0.04 D, which was far lower than the toric IOL 0.5 D gradation of cylinder power at the cornea plane.

Another interesting finding was the significant difference between A-ACS and TCA, both in IOLMaster and Pentacam. Since it is not easy to measure the total cornea curvature, using and calculation of toric and presbyopia correcting IOLs were based on the data obtained from manual keratometry and other devices. These data are not the total keratometry, sometimes leading to significant postoperative refractive errors [34]. Pentacam is a good method to obtain total corneal astigmatism data, but sometimes ophthalmologists need another device/method to identify the difference of astigmatism between the anterior and posterior cornea. Previous studies have shown that the significant differences of astigmatism are associated with the posterior corneal surface. This study also revealed significant differences between the astigmatism of anterior and total cornea, both by using IOLMaster and Pentacam. Our finding made an evidence to support that the curvature of the posterior cornea could be not ignored in ophthalmology clinic. Furthermore, measured with IOLMaster and Pentacam, the difference of anterior corneal surface (0.04 ± 0.45 D) was relatively larger than that of total keratometry (0.02 ± 0.58 D). What does it mean? We may need to collect more cases for further exploration. Our study showed that after vector analysis, the correct difference between A-ACS and TCA measured with IOLMaster was smaller than that of Pentacam. This difference might be due to the difference of measuring diameters as shown in Table 2. The measuring diameter of IOLMaster 700 is 2.5 mm for both anterior and total cornea. In Pentacam, the measuring diameter for the anterior surface is 3.0 mm, and that for the posterior is 4.0 mm. However, this reason could not explain the difference of IOLMaster 700 and Pentacam comparing A-ACS with TCA. A possible cause might be the effect of tear film on those different measurements [35–37]. But the advance study is still needed.

In conclusion, the present study evaluated the consistency of total corneal astigmatism measurement between IOLMaster and Pentacam. IOLMaster can accurately measure total keratometry. Moreover, the astigmatism difference between anterior corneal surface and total cornea was also identified, both of IOLMaster and Pentacam.

## Figures and Tables

**Table 1:** Demographics of included patients.

Characteristics	No.
Eyes (patients)	204 (102)
Age (years) (mean ± SD)	70.57 ± 8.95
Sex (% female)	64 (62.75%)
Axial length (mm) (mean ± SD)^∗^	23.77 ± 1.75
Anterior chamber depth (mm) (mean ± SD)^∗^	3.14 ± 0.64

SD = standard deviation. ^∗^Measured by IOLMaster.

**Table 2:** Corneal power and astigmatism obtained using IOLMaster and Pentacam.

	IOLMaster at 2.5 mm (mean ± SD) (range)	Pentacam at 3.0 mm (anterior corneal surface) or 4.0 mm (total cornea) (mean ± SD) (range)
K flat of anterior corneal surface, K1 (D)	44.16 ± 1.60 (38.62~49.91)	44.31 ± 1.57 (38.70~49.50)
K flat of anterior corneal surface axis (°)	2.16 ± 59.94 (-89.00~90.00)	1.88 ± 50.13 (-88.60~89.30)
K steep of anterior corneal surface, K2 (D)	45.09 ± 1.68 (41.55~53.63)	45.22 ± 1.65 (40.50~52.00)
Astigmatism of anterior corneal surface (A-ACS) (D)	−0.94 ± 0.63 (-4.84~0.00)	−0.90 ± 0.59 (-3.80~0.00)
K flat of total cornea, TK1 (D)	44.12 ± 1.62 (39.05~50.15)	44.15 ± 1.67 (40.00~49.00)
K flat of total cornea axis (°)	1.21 ± 64.34 (-89.00~90.00)	2.52 ± 55.61 (-88.90~89.90)
K steep of total cornea, TK2 (D)	45.14 ± 1.69 (41.63~54.13)	45.19 ± 1.82 (41.90~55.40)
Astigmatism of total cornea (TCA) (D)	−1.02 ± 0.69 (-4.76~0.00)	−1.05 ± 0.81 (-7.20~0.00)

D = diopters; K = keratometry.

## Data Availability

The data used to support the findings of this study are included within the article.
